# The Story of the Insane from Year to Year

**Published:** 1902-12-27

**Authors:** 


### THE STORY OF THE INSANE FROM YEAR TO YEAR.

FIFTEENTH ANNUAL SERIES.

### ROYAL ASYLUM, DUNDEE.

HERE the numbers were almost stationary, the figures showing an increase of 7 only during the year, and on December 31st 419 patients were in residence. There were 113 first admissions, 26 readmissions, 38 recoveries, 37 discharged relieved, and 54 died. The average number resident was 414, and the total number under treatment was 549. The recoveries stand at 27·34 per cent. of the admissions, and the deaths at 13 per cent. of the average number resident; the former percentage is low and the latter is high. Of the year's deaths, 22 were caused by cerebral and spinal diseases, 5 by consumption, 3 by pneumonia, and 1 by enteritis. Of the year's admissions the insanity was supposed to have been caused by moral causes, such as mental anxiety, religion and domestic trouble, in 45 instances. Hereditary influence accounts for 12, previous attacks for 31, and intemperance in drink for 47. The asylum contains about 85 private patients, and for these a new block has been recently opened. The rates for private patients vary from £40 to £180 per annum. The rate of maintenance for paupers is 12s. 6d. per head per week.

### MIDDLESEX COUNTY ASYLUM AT WANDSWORTH.

ON January 1st there were 1,314 patients in residence, and at the end of the year the number had risen to 1,414. There were 424 first admissions, 59 readmissions, 128 discharged recovered, 59 discharged relieved, 83 discharged not improved, and 113 died. The average daily number resident was 1,389, and the total number under treatment was 1,791. Calculated on the admissions the recoveries were 31·6 per cent., and calculated on the average number resident the deaths were 8·1 per cent. Of the year's deaths 46 were caused by cerebral and spinal diseases, 22 by consumption, and 8 by other lung diseases. Of the year's admissions 65 were supposed to owe their insanity to various moral causes, 41 to intemperance in drink, 80 to hereditary influences, 94 to previous attacks, and in 142 the cause was unknown. The asylum is about full, and contracts are in force at other asylums for the maintenance of 329 patients. The clerk of the asylum retired during the year, and was given the maximum superannuation permissible under the Lunacy Acts. It is pleasing to be able to record this liberality, and also that Nurse Agnes Cousens was treated in the same way. The committee refer to the "unflagging interest displayed by the medical superintendent." The weekly charge to the Unions was 12s. per head.

### HOSPITALS FOR THE INSANE IN VICTORIA, AUSTRALIA.

SEPARATE reports for these asylums do not reach us, but we are favoured with an official statement by the Government Inspector, and this statement contains much information in a small space. The asylum at Yarra Bend contained 791 patients, that at Kew contained 858, the Idiot Asylum there contained 267, that at Ararat 705, that at Beechworth 674, that at Sunbury 655, and that at Ballarat 356. There were at the close of the year many cases boarded out or out on probation, and, including these, there was a total of 4,501 registered lunatics in the colony. Lumping all these asylums together we find that there were 679 first admissions, 112 readmissions, 299 were discharged recovered, 27 discharged relieved, and 330 died. Calculated on the admissions the recoveries reach the fair percentage of 38·88, and calculated on the average number resident the deaths stand at 7·68 per cent. Table XII. shows that of the year's admissions 329 were natives of Victoria, 117 were English, 32 Scotch, 86 Irish, and 48 to other Australian colonies or to British Possessions. The total cost of maintenance of the lunatics in the Colony amounts to £127,763, but this sum includes general expenses and also expenses in connection with the committal and transport of the patients. The net cost per week per patient was 9s. 4¼d. The report states that efforts have been made to reduce the amount of restraint used in these asylums, and that they compare unfavourably with similar institutions in other countries; but we are not informed as to the numbers of patients who are restrained during the year. A table showing this and another showing the causes of death might be added in next year's report. Yet another, showing the causes of insanity in those admitted might find a place. As to restraint, it should be used just as often as it is the best treatment for the patient himself and for his fellow-patients. That it is possible to do without restraint altogether is undoubted; that it is desirable to so abolish it has never been proved to us. Dr. McCreery proposes to submit a scheme whereby patients suffering from tubercular disease may be removed from the general asylums, and placed in cheap, temporary buildings at Sunbury. We look upon this as a most desirable measure, and we congratulate Victoria on being able to solve a difficulty which taxes our wits in the old country. It is a deplorable fact that mental disease must be considerably advanced before a medical practitioner dare sign a certificate which would enable the patient to be treated at an early stage, and it is precisely at this early stage that treatment is so desirable and so successful. To prevent this delay a receiving house is to be built in Victoria, but Dr. McCreery thinks this will only be a partial remedy, and he recommends the establishment of special wards in connection with large general hospitals. Salaries have been increased, and attendants now begin at £66 per annum, and nurses at £35. The inspector states that the Victorian lunatic asylums are in many ways below the modern standards, and he attributes the present unsatisfactory state of affairs to absence of proper authority, to division of what authority there is, and to the absence of individual responsibility. All these are very serious things, but all should be easily remedied, and we should say that all three might vanish by a stroke of the pen which made the medical superintendent all-powerful in the asylum, and responsible to the full extent of that power. These two, power and responsibility, must always be united if the institution is to be a distinct success, and in fact they constitute success in a vast majority of cases. If failure follows, the culprit is easily found. The inspector continues: "Having thought it my duty to draw attention to the existing faults in our asylums, it is only fair to the officers and staff generally of these institutions to point out that good work has been done in many directions."

### GLOUCESTER COUNTY ASYLUMS.

ON January 1st these asylums contained 1,067 patients, and on December 31st the number had decreased to 1,017, but this decrease was owing to the removal of out-county patients to their own asylums, and not to any lack of lunatics in the county of Gloucester. There were 203 first admissions, 33 readmissions, 60 discharged recovered, 13 discharged relieved, and 106 died. The average daily number resident was 1,072. The recoveries were 27·14 per cent. of the admissions, and the deaths were 9·88 per cent. of the average number resident. Of the year's deaths, 38 were caused by cerebral and spinal diseases, 12 by consumption, and 4 by colitis. Of the year's admissions, 51 were supposed to owe their insanity to domestic trouble, grief, religion, and other moral causes. Among the physical causes hereditary influence, alone or in combination with other causes, stands first with 29, and intemperance in drink with 26. When dealing with the mortality table, Dr. Craddock refers specially to colitis, and he says "it appears difficult to guard against its outbreaks, and it is just as likely to occur, and has occurred, in a newly built and opened asylum as in an old place like ours." This agrees exactly with our own observations, and we believe, with Dr. Craddock, that scrupulous cleanliness and disinfection are the best prophylactics. He further thinks "that isolation is futile." We are not quite satisfied that this impression is right; but neither shall we dogmatise. Dr. Craddock's report is never dull reading, and we could wish that space in our columns permitted of long extracts. He believes firmly that insanity is increasing among us, and the utter disregard with which the governing classes look on this ever increasing burden finds expression as follows:—"If it cannot be diminished, in the name of common sense, humanity, and prudent forethought, let us try to prevent its increase. But how? Occurring insanity is not to be cured by elaborate Acts of Parliament . . . it is not going to be stayed by histological and bacteriological research . . . in a word, insanity is not to be cured, it must be prevented. Perhaps when our august legislature can spare time from futile discussions as to which particular scheme of education can render children least unfit to become useful citizens; when our religious pastors and masters have settled which is the most direct route to Heaven, and one or two pressing social questions have been disposed of, it might be worth while considering whether anything can be done to scotch, if not to destroy, the fell malady which is slowly eating into the very heart of the nation, and by so doing lighten the heavy financial burden imposed by the baleful growth and ever-increasing spread of the upas tree of lunacy." We fear it is too much to hope that these words will produce any effect; and yet constant dropping of water will wear away the hardest stone, and the evangel should be preached by every medical superintendent in England. The ever-recurring changes among the staff of attendants and nurses are fruitful sources of complaint by asylum managers. "It is a great mistake," Dr. Craddock says, "to take attendants from other asylums, and I never do so wittingly." This is an admirable rule, and adherence to it would tend more than almost anything else to secure that permanent service which all superintendents wish for in the staff. It is a good index of the quality of the subordinate staff, and to all those who think of becoming attendants and nurses we say, "Do not apply at any asylum where cast-offs from other asylums are engaged." The visitors say that Dr. Craddock "has the entire confidence of the committee." The weekly charge for Gloucestershire patients is 9s. Private patients pay from 15s. to 20s. a week.

